# Rapid Sediment Accumulation Results in High Methane Effluxes from Coastal Sediments

**DOI:** 10.1371/journal.pone.0161609

**Published:** 2016-08-25

**Authors:** Matthias Egger, Wytze Lenstra, Dirk Jong, Filip J. R. Meysman, Célia J. Sapart, Carina van der Veen, Thomas Röckmann, Santiago Gonzalez, Caroline P. Slomp

**Affiliations:** 1 Department of Earth Sciences–Geochemistry, Faculty of Geosciences, Utrecht University, Utrecht, The Netherlands; 2 Department of Estuarine and Deltaic Studies, Royal Netherlands Institute for Sea Research, Yerseke, The Netherlands; 3 Department of Analytical, Environmental, and Geochemistry, Vrije Universiteit Brussel, Brussels, Belgium; 4 Institute for Marine and Atmospheric Research Utrecht, Utrecht University, Utrecht, The Netherlands; 5 Laboratoire de Glaciologie, Université Libre de Bruxelles, Brussels, Belgium; 6 Department of Marine Microbiology and Biogeochemistry, Royal Netherlands Institute for Sea Research (NIOZ), Texel, The Netherlands; Griffith University, AUSTRALIA

## Abstract

Globally, the methane (CH_4_) efflux from the ocean to the atmosphere is small, despite high rates of CH_4_ production in continental shelf and slope environments. This low efflux results from the biological removal of CH_4_ through anaerobic oxidation with sulfate in marine sediments. In some settings, however, pore water CH_4_ is found throughout the sulfate-bearing zone, indicating an apparently inefficient oxidation barrier for CH_4_. Here we demonstrate that rapid sediment accumulation can explain this limited capacity for CH_4_ removal in coastal sediments. In a saline coastal reservoir (Lake Grevelingen, The Netherlands), we observed high diffusive CH_4_ effluxes from the sediment into the overlying water column (0.2–0.8 mol m^-2^ yr^-1^) during multiple years. Linear pore water CH_4_ profiles and the absence of an isotopic enrichment commonly associated with CH_4_ oxidation in a zone with high rates of sulfate reduction (50–170 nmol cm^-3^ d^-1^) both suggest that CH_4_ is bypassing the zone of sulfate reduction. We propose that the rapid sediment accumulation at this site (~ 13 cm yr^-1^) reduces the residence time of the CH_4_ oxidizing microorganisms in the sulfate/methane transition zone (< 5 years), thus making it difficult for these slow growing methanotrophic communities to build-up sufficient biomass to efficiently remove pore water CH_4_. In addition, our results indicate that the high input of organic matter (~ 91 mol C m^-2^ yr^-1^) allows for the co-occurrence of different dissimilatory respiration processes, such as (acetotrophic) methanogenesis and sulfate reduction in the surface sediments by providing abundant substrate. We conclude that anthropogenic eutrophication and rapid sediment accumulation likely increase the release of CH_4_ from coastal sediments.

## 1. Introduction

In most marine sediments, methane (CH_4_) is efficiently converted to carbon dioxide (CO_2_) by anaerobic oxidation coupled to sulfate (SO_4_^2-^) reduction within a distinct sulfate/methane transition zone (SMTZ) [1–5]. This removal of pore water CH_4_ in the SMTZ functions as an important sink for oceanic CH_4_ and prevents the large amount of CH_4_ generated in marine sediments from escaping to the water column [1,2,6,7]. As a consequence, the ocean provides only a relatively small contribution to the accumulation of this potent greenhouse gas in the atmosphere. In addition, the high affinity of SO_4_^2-^ reducing bacteria to hydrogen [8] and acetate [9] allows them to successfully outcompete methanogens for these common substrates. Hence, CH_4_ typically does not accumulate in the pore water of marine sediments until they become depleted in dissolved SO_4_^2-^.

Current knowledge suggests that anaerobic oxidation of methane (AOM) is most likely performed through a syntrophic relationship between methanotrophic archaea and sulfate reducing bacteria [1–4] and may involve various possible cooperative metabolic strategies [3,10–15]. However, the relevant metabolic pathways and the environmental factors that control the rates of AOM are still incompletely understood.

In particular, observations of large overlaps between pore water CH_4_ and SO_4_^2-^, with CH_4_ even tailing up to the sediment surface, suggest that AOM forms an inefficient oxidation barrier in certain marine environments [16–22]. Such a sluggish microbial turnover of pore water CH_4_ may increase the CH_4_ release to the water column in case of rapid production of CH_4_ in the sediments, for example as a result of destabilization of temperature-sensitive clathrate reservoirs [23] or increased organic matter deposition due to anthropogenic eutrophication [24]. To date, the reasons for this apparent inefficiency in CH_4_ removal with SO_4_^2-^ remain largely unknown.

In sediments of the Black Sea, where CH_4_-tailing appears to be a common pattern, the sluggish CH_4_ oxidation could not be related to unusual low rates of AOM or the lack of methanotrophic organisms [16,22,25]. The slow growth of CH_4_ oxidizing communities [23,26] does indicate that methanotrophs may have difficulties in keeping up with high rates of sediment accumulation, such as found in near shore and estuarine environments [17]. High sediment accumulation rates in these coastal systems typically result in a relatively short residence time of organic matter in the SO_4_^2-^ reduction zone, allowing for extensive CH_4_ production deeper in the sediment [2,17,27–30]. As a consequence, near shore environments are responsible for a major part of oceanic CH_4_ emissions [2,30–32]. However, little is known about the impact of anthropogenic eutrophication on CH_4_ dynamics in coastal systems.

Besides causing the development of “dead zones”, i.e. coastal waters subject to oxygen depletion (hypoxia) [24,33], anthropogenic eutrophication also impacts the sedimentary redox balance and associated biogeochemical processes [34]. Hypoxia can thus result in a shoaling of the biogeochemical zonation in the sediments [34], allowing for CH_4_ production in more shallow sediments and an enhanced CH_4_ flux to the bottom water [31]. In addition, increased input of organic matter to the sediment can induce a vertical upward migration of the SMTZ in coastal sediments through enhanced rates of SO_4_^2-^ reduction and methanogenesis [35–37], shifting the CH_4_ oxidation barrier closer to the sediment surface. The development of bottom water hypoxia due to enhanced nutrient loading combined with the potential for limited CH_4_ removal as a result of fast sediment accumulation may therefore greatly increase atmospheric CH_4_ emissions from the coastal ocean.

In this study, we use detailed geochemical analyses as well as reactive transport modeling of the sediment and pore water of cores collected from a seasonally hypoxic coastal basin in the Netherlands to demonstrate how rapid sediment accumulation in combination with high organic matter loading impact early diagenesis in coastal marine sediments. The study area was selected because of its recent history of eutrophication and the known high rates of sediment accumulation in the region (e.g. [38]). Our results reveal that CH_4_ bypasses the SO_4_^2-^ reduction zone in this high sedimentation rate environment. The subsequent lack of removal through AOM in combination with CH_4_ production close to the sediment surface allows a high CH_4_ efflux from the sediment to the water column to be sustained, thus increasing the potential of CH_4_ escaping to the atmosphere. We further show that SO_4_^2-^ reduction, methanotrophy, methanogenesis and Fe oxide reduction likely co-occur in marine sediments with high rates of sediment accumulation.

## 2. Methods

### 2.1 Study location

**Fig 1:**
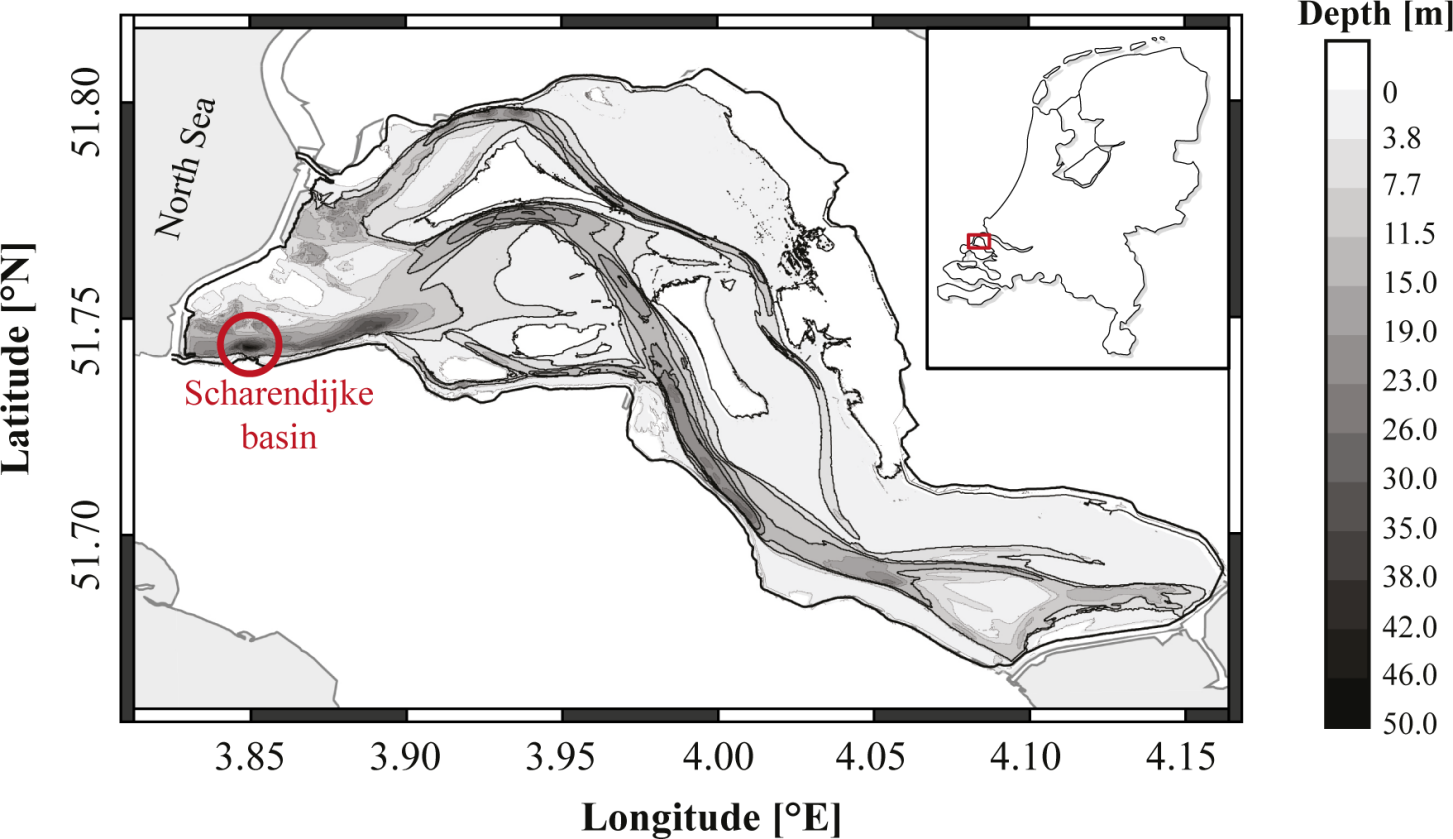
Bathymetric map of marine Lake Grevelingen. Cores were taken in the Scharendijke basin (51.742°N, 3.849°E; red circle) between 2012 and 2015. The red rectangle indicates the location of Lake Grevelingen in The Netherlands (NL).

Sediment cores were collected from the Scharendijke basin (51.742°N, 3.849°E) in Lake Grevelingen, a saline coastal reservoir in the Netherlands (Fig 1). Sampling occurred during multiple sampling campaigns on the R/V Luctor and R/V Navicula between 2012 and 2015 (Table A in S1 File). Before the construction of two dams in 1964 and 1971, Lake Grevelingen was an estuarine ecosystem. After enclosure, it became a seasonally stratified saline reservoir (salinity ~ 29–32), where bottom water oxygen depletion develops each summer in the deeper basins of the former estuarine tidal channels [39]. A detailed description of the hydrodynamics, biogeochemistry and development of hypoxia within Lake Grevelingen is given in [40]. The sampling site (water depth 45 m) is located in the central part of the Scharendijke basin, which is the deepest basin in Lake Grevelingen. Note that all sampling occurred during oxic bottom water conditions, i.e. between the months of October and May.

### 2.2 Core sampling and pore water collection

Sediment cores were collected using a UWITEC gravity corer with transparent PVC core liners of either 60 or 120 cm length (inner diameter 6 cm). Pore water was extracted immediately on recovery, either by centrifugation of sliced sediment samples or using rhizons ([41]; Rhizosphere Research Products). An overview of the different sampling methods applied during the various sampling campaigns is given in Table A in S1 File.

Upon recovery, one core was inserted into a nitrogen (N_2_)-purged glove bag through an airtight hole in the base. A bottom water sample was collected using a 20 mL plastic syringe positioned in the overlying water ~ 5–10 cm from the sediment surface, and the remaining bottom water was removed. The core was then sliced under an inert atmosphere at 0.5 cm resolution for the first 0–2 cm, 1 cm resolution for 2–10 cm, and 2 cm for the rest of the core (> 10 cm) in November and December 2012, at 1 cm resolution for the first 0–5 cm, 2.5 cm resolution for 5–20 cm, and 4 cm for the rest of the core (> 20 cm) in February 2013, and at 1 cm resolution for the whole core in May 2015, respectively, using a push-up pole to extrude the sediment from the PVC core liner. For each slice a sub-sample was placed in a pre-weighed glass vial to determine sediment porosity (calculated from the weight loss after freeze-drying assuming a sediment density of 2.65 g cm^-3^) and for solid phase analysis and stored under N_2_ in airtight jars at -20°C. A second sub-sample was transferred to a 50 mL centrifuge tube and centrifuged at 4500 rpm for 15 min. Both the supernatant water from each centrifuged sample and the bottom water sample were filtered through 0.45 μm pore size disposable filters via 20 mL plastic syringes in a glove bag under N_2_ and collected in 15 mL centrifuge tubes. Filtered pore water samples, including the bottom water sample, were sub-sampled under N_2_ for analysis of pore water ammonium (NH_4_^+^), Fe, SO_4_^2-^ and dissolved sulfide ([∑H_2_S] = [H_2_S] + [HS^-^]) as described in section 2.3 below.

### 2.3 Pore water sub-sampling

A pore water sub-sample of 0.5 mL was immediately transferred into a glass vial (4 mL) containing 2 mL of 2% zinc (Zn)-acetate solution to precipitate ZnS, which was stored at 4°C. Sulfide concentrations were determined spectrophotometrically by complexion of the ZnS precipitate in an acidified solution of phenylenediamine and ferric chloride [42]. The detection limit was ~ 1 μmol L^-1^ and the sulfide standard was validated by titration with thiosulfate. Samples for NH_4_^+^ were stored frozen at -20°C until colorimetric determination on a nutrient auto-analyzer (Bran and Luebbe). Sub-samples for total dissolved S and Fe, which are assumed to represent SO_4_^2-^ and Fe^2+^, were acidified with 10 μL 35% suprapur HCl per mL of sub-sample and stored at 4°C until analysis by ICP-OES (Perkin Elmer Optima 3000 Inductively Coupled Plasma—Optimal Emission Spectroscopy).

Diffusive fluxes (*J*_*i*_, in mol cm^-2^ yr^-1^) of pore water constituents were calculated as:

$$J_{i}=\phi \frac{D_{i,sw}}{1-2ln\phi }\frac{\Delta C_{i}}{\Delta x}$$ (1)

where ϕ is the measured porosity (cm^3^ cm^-3^), *D*_*i*,*sw*_ is the molecular diffusion coefficient for solute *i* in seawater (cm^2^ yr^-1^), calculated as a function of the in situ temperature, pressure and salinity [43] as measured in the bottom water using a YSI6600 CTD probe [40], C is the concentration of substance *i* in mol cm^-3^, and *x* is the sediment depth in cm.

### 2.4 Methane sampling and analysis

Sediment samples for CH_4_ analysis were taken directly upon core retrieval through pre-drilled holes (diameter 2 cm; 5 cm vertical resolution) that were taped prior to coring. Precisely 10 mL of wet sediment was extracted from each hole and immediately transferred into a 65 mL glass bottle pre-filled with a saturated NaCl solution. The solution was topped up after addition of the sample, ensuring that no air bubbles entered the bottle. The bottle was sealed with a black rubber stopper and a screw cap and subsequently stored upside-down. Prior to analysis, a volume of 10 mL N_2_ was injected into the bottle (while a needle inserted through the rubber stopper allowed 10 mL of solution to escape) to create a headspace from which a sub-sample was collected with a gas-tight syringe after headspace equilibration. Subsequently, CH_4_ concentrations were determined under laboratory conditions by injection into a Thermo Finnigan Trace GC gas chromatograph (Flame Ionization Detector). δ^13^C-CH_4_ and δD-CH_4_ (D, deuterium) were analyzed by Continuous Flow Isotope Ratio Mass Spectrometry (CF-IRMS) as described in detail in [36,44,45].

### 2.5 Sulfate reduction measurements

During the sampling campaign in November 2015, sediment samples for SO_4_^2-^ reduction rate measurements were taken from two replicate cores, which were pre-drilled (diameter 2 cm; 5 cm vertical resolution) and taped prior to coring. The taped holes were cut open directly upon core retrieval and 5 mL of wet sediment was extracted using cut-off syringes. Subsequently, the syringes were sealed with parafilm that was tightly closed with an elastic band and stored under an inert nitrogen (N_2_) atmosphere in the dark at 4°C.

Within 24 hours of coring, a volume of 20 μL carrier-free ^35^SO_4_^2-^ (42.4 kBq) was injected in the syringes using a micropipette with a needle on top (while pulling the pipette out of the sample to equally distribute the ^35^SO_4_^2-^ over the whole syringe). After injection, the hole made by the needle was sealed by a second layer of parafilm and closed with an elastic band. The sediment was incubated for 20 h in the dark at 4°C (under inert N_2_ atmosphere) before it was transferred to a 50 mL centrifuge tube containing 20 mL oxygen-free 20% Zn-acetate to precipitate dissolved sulfide and terminate microbial activity [46–48]. Centrifuge tubes were subsequently stored frozen (-20°C) under N_2_. Upon analysis, samples were washed two times with oxygen-free bottom water (10 mL) and centrifuged to remove pore water and unreacted ^35^SO_4_^2-^. The reduced S was determined by extraction with an acidic chrome chloride solution for 48 h via the passive diffusion method described by [49]. Subsequently, SO_4_^2-^ reduction rates (SRR) were calculated by comparing the activity (decays per minute) of the radiolabeled total reduced inorganic sulfur (*a*_*TRIS*_) to the total SO_4_^2-^ radiotracer (*a*_*TOT*_) as described in [48]:

$$SRR=[SO_{4}]\times \phi \times \frac{a_{TRIS}}{a_{TOT}}\times \frac{1}{t}\times 1.06$$ (2)

where *ϕ* is the measured porosity (to correct for pore water volume), *t* is the incubation time in days and 1.06 is the correction factor for the expected isotopic fractionation [48,50]. The potential contribution of unreacted ^35^SO_4_^2-^ to the SRR measurement was estimated to be < 0.1%.

### 2.6 Solid phase analysis

Sediment samples were freeze-dried, powdered and ground in an agate mortar inside an argon (Ar)-filled glove box and split into two fractions. Samples from the first fraction were stored under normal (i.e. oxic) atmospheric conditions and used for total elemental and organic carbon (C_org_) analyses. The second fraction was used for sediment Fe speciation and kept under an inert, oxygen-free Ar or N_2_ atmosphere at all times to avoid oxidation artefacts.

#### 2.6.1 Total elemental composition and organic carbon

A portion of ~ 125 mg of freeze-dried sediment was dissolved overnight in 2.5 mL HF (40%) and 2.5 mL of HClO_4_/HNO_3_ mixture, in a closed Teflon bomb at 90°C. The acids were then evaporated at 160°C (not to complete dryness) and the resulting gel was dissolved overnight in 1 M HNO_3_ at 90°C. Total elemental concentrations in the 1 M HNO_3_ solutions were determined by ICP-OES. A second split of ~ 0.3 g freeze-dried sediment was used to determine the C_org_ content using an elemental analyzer (Fison Instruments model NA 1500 NCS) after carbonate removal from the sediment with two washes with 1 M HCl (4 h and 12 h) followed by two washes with UHQ (Ultra High Quality) water and subsequent drying of the samples [51].

For simplicity, only solid phase data for May 2015, of which the most exhaustive dataset is available, are shown in the main text. Additional solid phase data for November 2012, December 2012 and February 2013 are presented in Figure A in S1 File and generally compare well with data from May 2015 when accounting for temporal changes in bottom water conditions and sediment deposition.

#### 2.6.2 Sediment Fe fractionation

Sediment Fe was fractionated into i) carbonate associated Fe (“Fe_carb_”, including siderite and ankerite, extracted by 1 M Na-acetate brought to pH 4.5 with acetic acid, 24 h), ii) easily reducible (amorphous) oxides (“Fe_ox1_”, including ferrihydrite and lepidocrocite, extracted by 1 M hydroxylamine-HCl, 24 h), iii) reducible (crystalline) oxides (“Fe_ox2_”, including goethite, hematite and akagenéite, extracted by Na-dithionite buffer, pH 4.8, 2 h) and iv) Fe in recalcitrant oxides (mostly magnetite, “Fe_magn_”, extracted by 0.2 M ammonium oxalate / 0.17 M oxalic acid solution, 2 h), according to [52], using a ~ 50 mg aliquot of dried sediment.

A third 0.5 g aliquot of dried sediment was used to sequentially determine the amount of FeS (acid volatile sulfur, AVS, using 6M HCl) and FeS_2_ (chromium reducible sulfur, CRS, using acidic chromous chloride solution) via the diffusion-based approach described by Burton et al. (2008) using iodometric titration of the ZnS formed in the alkaline Zn-acetate traps to quantify AVS and CRS.

### 2.7 Reactive transport modeling

To gain a better quantitative understanding of the CH_4_ cycling at our study site, a simplified version of a previously developed 1-dimensional reactive transport model was applied [35,53]. The model describes the cycling of 14 particulate and dissolved chemical species (Table B in S1 File) in the upper 100 cm of sediment (0.5 mm vertical resolution) via a set of mass conservation equations, which include transport processes as well as biogeochemical transformations [43,54,55]:

$$(1-\phi )\frac{\partial C_{S}}{\partial t}=-(1-\phi )v\frac{\partial C_{S}}{\partial x}+\sum R_{S}$$ (3)

$$\phi \frac{\partial C_{aq}}{\partial t}={\phi D}^{\prime }\frac{{\partial^{2}C}_{aq}}{\partial x^{2}}-\phi u\frac{\partial C_{aq}}{\partial x}+\sum R_{aq}$$ (4)

where *ϕ* is the sediment porosity (volume of pore water per volume of total sediment), *C*_*S*_ the concentration of the solid species (mol L^-1^; mass per unit volume of solids), *C*_*aq*_ the concentration of the dissolved species (mol L^-1^; mass per unit volume of pore water), t is time (yr), x the distance from the sediment-water interface (cm), *D*′ the diffusion coefficients of dissolved species in the sediment (cm^2^ yr^-1^) at in situ conditions and corrected for the tortuosity in the porous medium [56] (Table C in S1 File). ∑ *R*_*S*_ and ∑ *R*_*aq*_ are the net reaction rates of the solid and dissolved species from the chemical reactions they participate in (Table D in S1 File), and *v* and *u* the advective velocities (cm yr^-1^) of the solid and the dissolved species, respectively. Depth-dependent functions were used for porosity and advective velocities to account for sediment compaction [57,58] (Table C and Figure B in S1 File).

Reaction equations and corresponding reaction parameters implemented in the model are given Tables E and F in S1 File. Model boundary conditions are shown in Table G in S1 File. The model code was written in R (version 3.2.4) using the *marelac* geochemical dataset package [59] and the *ReacTran* package [60] to calculate the transport terms. Upon discretization of the mass conservation equations, the set of equations was solved with the lsoda ordinary differential equation solver [61] and the model was run to steady state. For a detailed model description, the reader is referred to Egger et al. (2016).

## 3. Results

### 3.1 Pore water profiles

**Fig 2:**
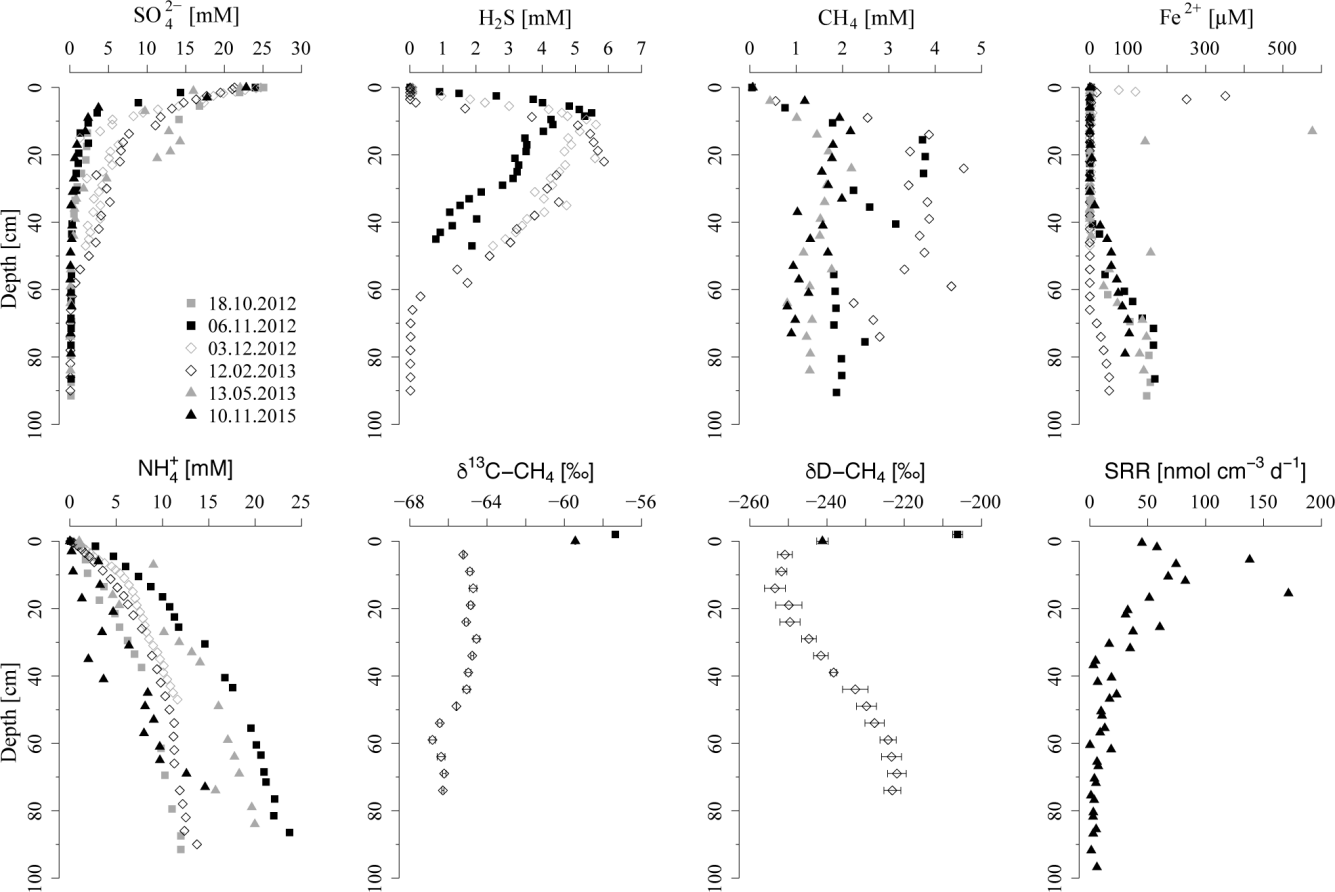
Pore water profiles of key components, as well as isotopic composition of pore water CH_4_ and measured SO_4_^2-^ reduction rates (SRR) in sediments of the Scharendijke basin. δ^13^C-CH_4_ values are given in ‰ vs. VPDB (Vienna Pee Dee Belemnite) and δD-CH_4_ values are given in ‰ vs. V-SMOW (Vienna Standard Mean Ocean Water). Note that bubble formation and degassing of CH_4_ was observed during coring, indicating that actual CH_4_ concentrations likely are higher.

Concentrations of SO_4_^2-^ show a rapid decrease from ~ 25 mM at the sediment-water interface to ~ 1–10 mM within the first 15 cm of sediment depth (Fig 2). This decrease is accompanied by a steep increase of dissolved sulfide from < 0.1 mM near the surface to > 5 mM in the same zone. Pore water sulfide reaches a maximum at ~ 15–20 cm and is detected down to a depth of ~ 60 cm, but SO_4_^2-^ also remains at low but detectable levels (> 0.08 mM) within the depth horizon from 20 to 60 cm.

Measured concentrations of pore water CH_4_ vary between 1–5 mM throughout most of the core, but increase rapidly in the upper 10 cm from 50–100 μM at the sediment-water interface to > 1 mM at depth in the sediment. Note that extensive bubble formation and degassing was observed during coring, suggesting that actual CH_4_ concentrations likely are higher. Dissolved Fe^2+^ profiles show distinct maxima of ~ 100 μM and ~ 350 μM around a depth of 1 cm and 2.5 cm in December 2012 and February 2013, respectively. At depths of 40 to 60 cm, pore water Fe^2+^ increases again to at most ~ 200 μM near 90 cm. Vertical profiles of NH_4_^+^ show a gradual increase to ~ 10–25 mM at depth.

The isotopic composition of pore water CH_4_ is depleted in ^13^C, showing values of δ^13^C-CH_4_ around ~ -66 ‰ below 50 cm depth and of ~ -65 ‰ in the upper 50 cm of sediment (Fig 2). In the bottom water samples, δ^13^C-CH_4_ increases to ~ -57 ‰. The values of δD-CH_4_ decrease from -220 ‰ at depth to ~ -250 ‰ close to the sediment surface, and increase again to ~ -206 ‰ in the bottom water. Measured SRR show highest rates of ~ 50–150 nmol cm^-3^ d^-1^ in the upper 20 cm of sediment and a subsequent gradual decrease to < 10 nmol cm^-3^ d^-1^ below 60 cm, resulting in an estimated areal SRR of ~ 9.2 mol m^-2^ yr^-1^.

### 3.2 Solid phase records

**Fig 3:**
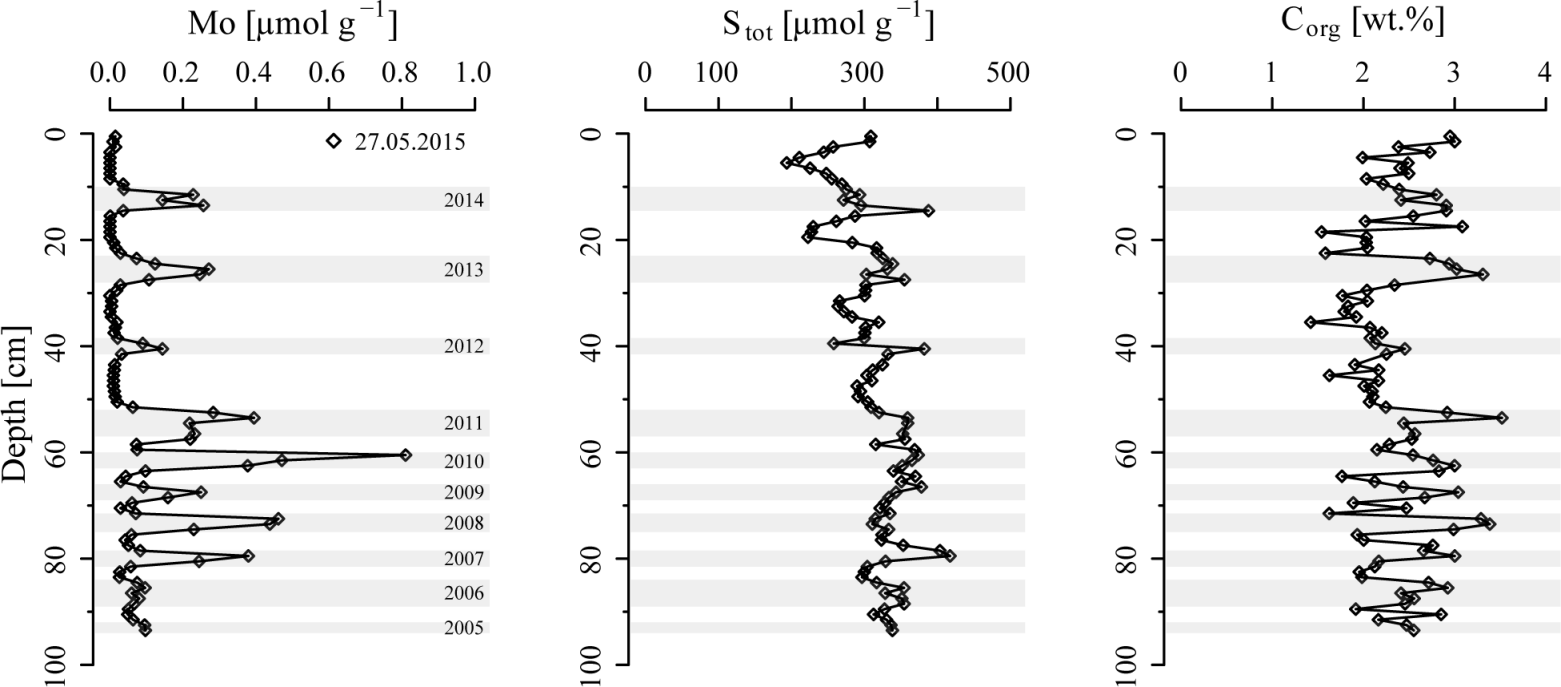
Vertical profiles of sedimentary Mo, total solid phase S and C_org_. Grey bars indicate sediments that are enriched in Mo relative to the background, which is assumed to reflect periods of seasonal hypoxia in Lake Grevelingen.

The sediments at the field site reveal distinct periodic peaks in solid phase molybdenum (Mo), with a characteristic periodicity of ~ 13 cm in the upper 50 cm of sediment, which decreases to ~ 7 cm at depth (Fig 3). The total solid phase S profile shows a slightly increasing trend with depth until ~ 50 cm and peaks in S generally correlate well with those in sedimentary Mo. Concentrations of C_org_ vary around ~ 2 to 3 wt.% throughout the whole core with maxima that coincide with peaks in Mo but no other discernible trend with depth (see also Figure A in S1 File).

**Fig 4:**
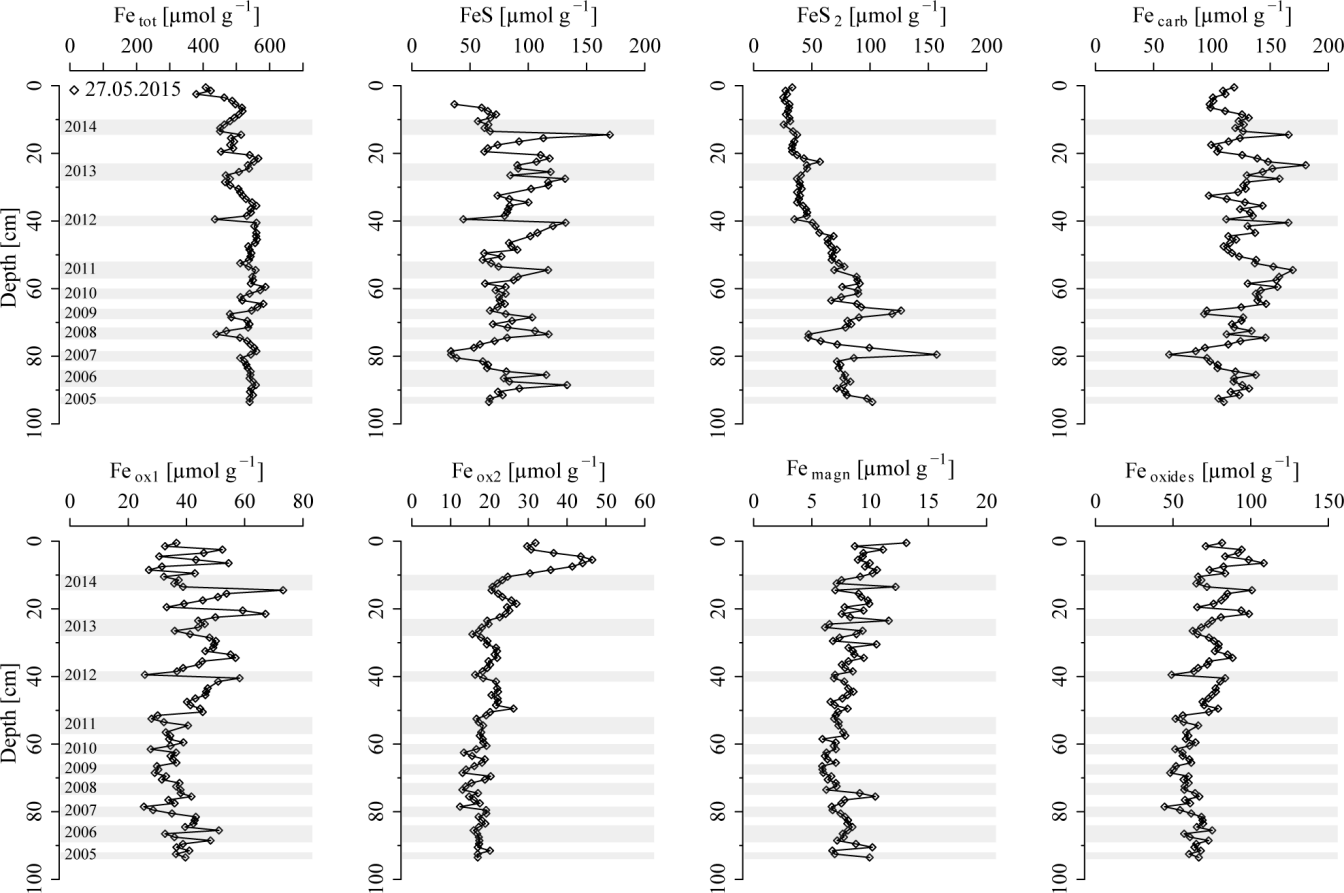
Fe extraction results including total sedimentary Fe (Fe_tot_), acid volatile sulfide (FeS), chromium reducible sulfur (FeS_2_), carbonate associated Fe (Fe_carb_), easily reducible (amorphous) oxides (Fe_ox1_), reducible (crystalline) oxides (Fe_ox2_), recalcitrant oxides (mostly magnetite, Fe_magn_) and total sedimentary Fe oxides (sum of Fe_ox1_, Fe_ox2_ and Fe_magn_). Note that the total amount of Fe oxides may be overestimated due to dissolution of FeS in the hydroxylamine-HCl step (Fe_ox1_) [36]. Also note the different scales on the x axes. Grey bars correspond to sediments with elevated concentrations of solid phase Mo, indicative for anoxic bottom waters in the Scharendijke basin during summer.

Total sedimentary Fe concentrations are around 400 μmol g^-1^ at the sediment surface, below which they rapidly increase to > 500 μmol g^-1^ (Fig 4). Sedimentary FeS varies between ~ 50 to 150 μmol g^-1^, while FeS_2_ increases with depth from ~ 50 μmol g^-1^ to > 100 μmol g^-1^. Concentrations of Fe associated with Fe carbonates and easily reducible (amorphous) oxides (Fe_ox1_) vary between ~ 100 to 200 μmol g^-1^ and ~ 20 to 70 μmol g^-1^, respectively, and generally correlate well with concentrations of FeS. Reducible (crystalline) Fe oxides (Fe_ox2_) show a subsurface peak of ~ 50 μmol g^-1^ around 5 cm and decrease to ~ 20 μmol g^-1^ at depth. The concentration of recalcitrant oxides (mostly magnetite, Fe_magn_) is around 10 μmol g^-1^. Concentrations of total sedimentary Fe oxides (i.e. the sum of Fe_ox1_, Fe_ox2_ and Fe_magn_) remain above 50 μmol g^-1^ in the upper 100 cm of sediment.

### 3.3 Modeling results

**Fig 5:**
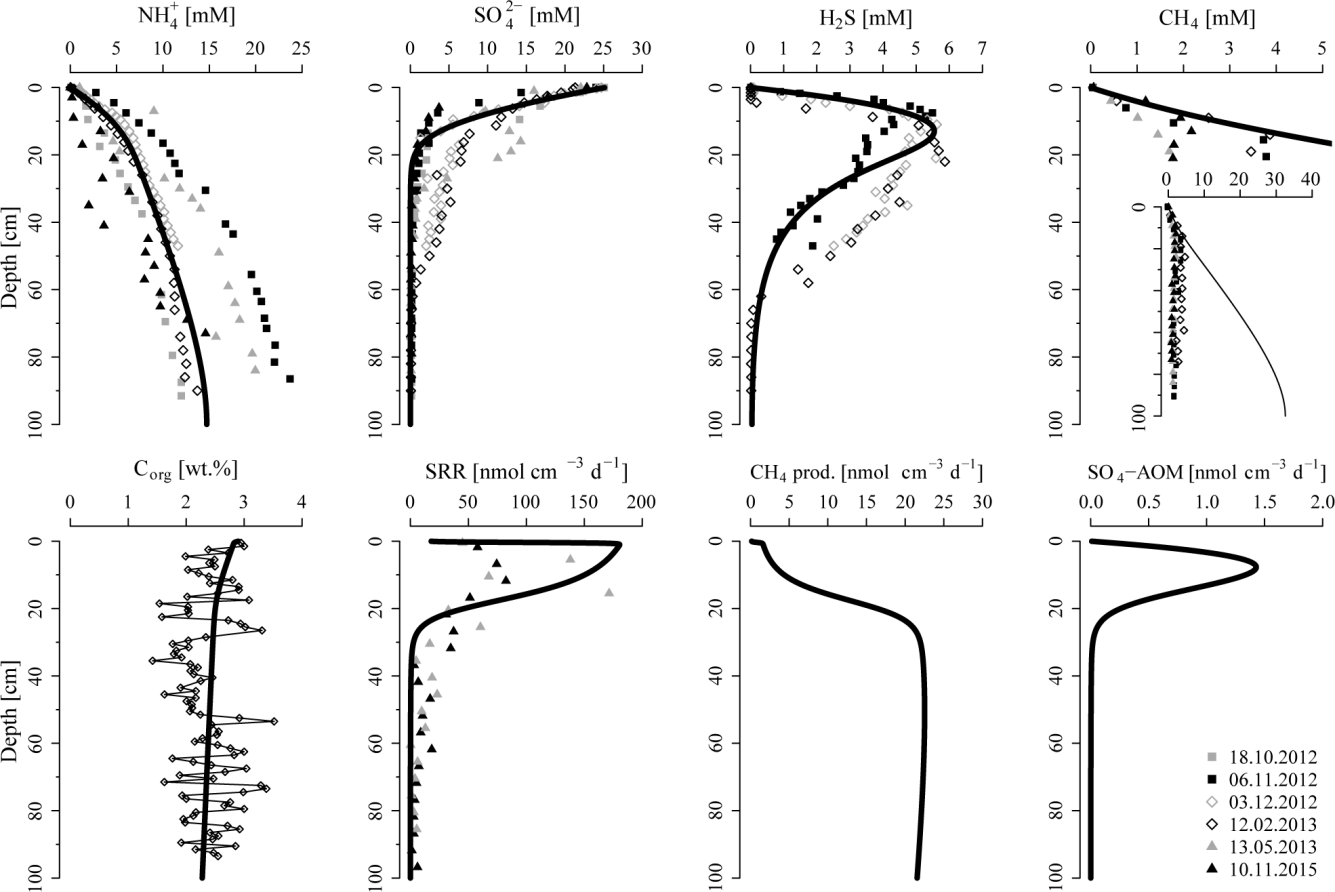
Profiles of selected pore water constituents and C_org_, as well as rates of SO_4_^2-^ reduction (SRR), methanogenesis and SO_4_-AOM derived from the reactive transport model. Note that extensive bubble formation and degassing of CH_4_ was observed during coring, explaining the offset between modeled and measured CH_4_ concentrations at depth. Also note that the modeled CH_4_ concentrations exceed the CH_4_ solubility concentrations of ~ 10 mM, indicating the presence of free CH_4_ gas in the sediments of the Scharendijke basin

The modelled pore water profiles of NH_4_^+^, SO_4_^2-^ and dissolved sulfide are in good agreement with the measured data (Fig 5). In addition, the model is able to reproduce the measured CH_4_ profile in the upper < 20 cm of sediment. However, while measured CH_4_ concentrations stay below ~ 5 mM, pore water CH_4_ accumulates to > 30 mM at depth in the model. Model-calculated concentrations of C_org_, which are also constrained by the NH_4_^+^ profiles, decrease from ~ 2.9 wt.% at the sediment surface to ~ 2.3 wt.% at depth, corresponding to a mineralization rate of ~ 20 mol C m^-2^ yr^-1^, an organic matter flux of ~ 91 mol C m^-2^ yr^-1^ at the sediment-water interface and ~ 71 mol C m^-2^ yr^-1^ at the bottom of the model domain, respectively. Modelled SRR show a broad maximum of ~ 170 nmol cm^-3^ d^-1^ within the upper 20 cm of sediment, consistent with estimated SRR based on ^35^SO_4_^2-^ radiotracer injection, and decrease to 14 pmol cm^-3^ d^-1^ at depth. Rates of methanogenesis increase from ~ 2 nmol cm^-3^ d^-1^ at 1 cm depth to ~ 20 nmol cm^-3^ d^-1^ below a depth of ~ 20 cm. Methane oxidation coupled to SO_4_^2-^ reduction displays highest rates of ~ 1.4 nmol cm^-3^ d^-1^ around 10 cm depth and declines to 0.28 pmol cm^-3^ d^-1^ at depth in the sediment.

Depth-integrated rates of oxic mineralization, denitrification, organoclastic Fe oxide reduction, organoclastic SO_4_^2-^ reduction and methanogenesis over the upper meter of the sediment are 1.95, 0.15, 0.39, 10.42 and 6.86 C mol m^-2^ yr^-1^, respectively. Thus, SO_4_^2-^ reduction and methanogenesis account for an estimated ~ 53% and ~ 35% of total organic matter degradation in sediments of the Scharendijke basin.

## 4. Discussion

### 4.1 Seasonal hypoxia and rapid sediment accumulation

Seasonal hypoxia is a well-documented phenomenon in Lake Grevelingen. Bottom waters are generally oxygenated from September to May, while hypoxic and/or anoxic conditions prevail below ~ 15 m water depth from early June to late August [38–40,62]. The major controls of the annual extent and intensity of hypoxia in the lake are temperature-induced summer stratification, as well as input of organic matter from the adjacent eutrophic North Sea [39]. In particular, intensive spring blooms of the marine phytoplankton *Phaeocystis* in the North Sea, influenced by anthropogenic eutrophication, are known to contribute to the carbon input to the lake and the increased oxygen demand in the deep basins of Marine Lake Grevelingen in spring and summer [39].

The Scharendijke basin forms the deepest basin in Lake Grevelingen, and so, it experiences the most intense and prolonged bottom water hypoxia [39]. Accordingly, one expects an accumulation of sedimentary Mo during summer due to the conversion of seawater MoO_4_^2-^ to particle-reactive oxythiomolybdates in the presence of H_2_S near the sediment-water interface [33,63–65]. The oscillations in sedimentary Mo (Fig 3) thus likely reflect seasonal cycles of anoxic bottom waters in the Scharendijke basin associated with summer stratification and water column hypoxia. Similar oscillations in solid phase Mo are observed in December 2012, but shifted by about ~ 32 cm in depth (Figure A in S1 File). The sedimentary Mo record suggests exceptionally high rates of sediment accumulation in the Scharendijke basin, with an abrupt increase from ~ 7 cm yr^-1^ to ~ 13 cm yr^-1^ around the year 2011. Based on the location of the study site in a deep basin with steeply inclined sides [39], it is likely that such rapid sediment accumulation is the result of lateral input and sediment focusing of material from both the North Sea and surrounding shallower areas of the lake.

**Fig 6:**
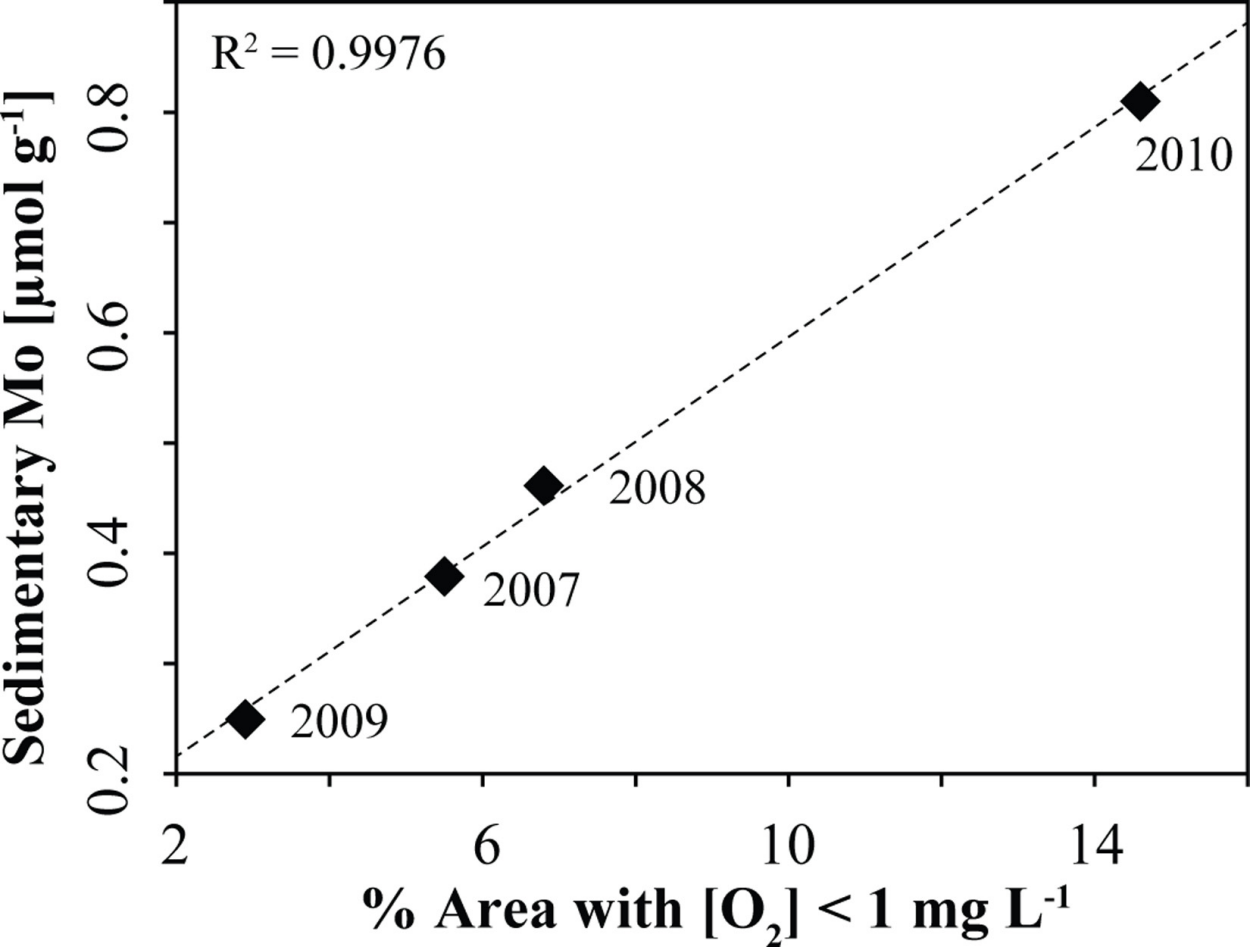
A comparison between the measured sedimentary Mo peak concentrations and the reported area of hypoxia for the years 2007 to 2010 [39] reveals a linear correlation (y = 0.0475x + 0.121).

Comparing the Mo peak concentrations with the reported area of hypoxia in Lake Grevelingen (sediment surface area exposed to bottom waters with [O_2_] < 31 μM) [39] reveals a good correlation between the reported areal extent of hypoxia (data only available until 2010) and sedimentary Mo enrichments (Fig 6). Using this linear correlation, we estimate an areal extent of hypoxia in Lake Grevelingen of 5.7%, 0.5%, 3.2% and 2.9% in the years 2011, 2012, 2013 and 2014, respectively. The lower estimate for the hypoxic area in 2012 is consistent with water column data obtained during monthly cruises in 2012 [38,40].

### 4.2 Organic matter burial and SO_4_^2-^ reduction

Rapid sediment accumulation and high concentrations of C_org_ reveal high rates of organic matter burial (~ 71 mol C m^-2^ yr^-1^) in sediments of the Scharendijke basin. This rate of organic matter burial is about an order of magnitude higher than typical rates of organic matter accumulation previously reported for estuarine sediments and due to the exceptionally high sediment accumulation rate. For example, [17] reported a rate of burial of 8.9–9.5 mol C m^-2^ yr^-1^ at a coastal site in the Baltic Sea with similar organic carbon content but ten times lower sedimentation rate (0.65–0.95 cm yr^-1^). The resulting short residence time of organic matter in the main SO_4_^2-^ reduction zone (< 5 years) at our site results in a burial efficiency of C_org_ of > 78%, which is also exceptionally high when compared to burial efficiencies in other continental margin settings (e.g. [66]). Concentrations of C_org_ vary between 2–3 wt.% in the upper 1 m of sediment, with the highest concentrations in sediments deposited during summer, i.e. in sediments with high Mo contents (Fig 3 and Figure A in S1 File). This is likely the result of the input of organic matter from spring blooms in the adjacent eutrophic North Sea [39], and from spring and summer blooms within Lake Grevelingen [40], combined with enhanced preservation of organic matter under bottom water hypoxia or anoxia in summer.

Pore water profiles of SO_4_^2-^ indicate that most of the SO_4_^2-^ reduction takes place in the upper 20 cm of sediment (Fig 2), consistent with highest measured and modeled SRR around 10 cm depth in the sediment (Fig 2 and Fig 5). Estimated areal SRR based on ^35^SO_4_^2-^ radiotracer injection (~ 9.2 mol m^-2^ yr^-1^) and reactive transport modeling (~ 10.4 mol m^-2^ yr^-1^) closely match the SRR estimated from the nearby Den Osse site within Lake Grevelingen (~ 11 mol m^-2^ yr^-1^) [62]. These rates fall at the high end of the range of reported average SRR for estuaries and high-deposition coastal environments (~ 2.6–13 mol m^-2^ yr^-1^) [67,68]. However, the estimated SRR are around a factor two higher than the diffusive fluxes of seawater SO_4_^2-^ into the sediments of the Scharendijke basin (1.9–4.5 mol m^-2^ yr^-1^). This difference between the estimated influx of SO_4_^2-^ into the sediment and measured areal SRR is likely due to the fact that diffusive fluxes represent net SO_4_^2-^ consumption, while SRR are a measure of total SO_4_^2-^ turnover [69]. Estimates based on pore water profiles of SO_4_^2-^ may thus underestimate the actual rate of SO_4_^2-^ reduction in marine sediments, as shown previously for example for Black Sea sediments [22,53].

### 4.3 Fe reduction in the methanogenic sediments

The high rates of SO_4_^2-^ reduction in the upper 20 cm of sediment lead to a release of sulfide to the pore water and, as a consequence, diffusion of dissolved sulfide towards the sediment surface and into the deeper sediment deposits (Fig 2). Pore water profiles of dissolved sulfide and Fe^2+^ available for February 2013 reveal that Fe^2+^ builds up at depth, as soon as pore waters become depleted in sulfide (< 30 μM). Thus, the increase in dissolved Fe^2+^ provides a reliable estimate for the sulfide penetration depth for sampling campaigns where no sulfide data are available, indicating that pore water sulfide is confined to the upper ~ 40 to 60 cm of sediment. This restriction of sulfide is due to the formation of FeS by reactions with sedimentary Fe oxides and dissolved Fe^2+^, as well as subsequent conversion to pyrite via the sulfide pathway (Table D in S1 File). The rapid sediment accumulation in the Scharendijke basin, however, reduces the exposure time of Fe oxides to dissolved sulfide, allowing for the burial of abundant reducible Fe oxides past the sulfidic zone (Fig 4) and their reduction at depth below the SO_4_^2-^-bearing zone (Fig 2).

To date, the underlying mechanism for the reduction of Fe oxides in methanogenic sediments remains enigmatic. The rapid burial of fresh organic matter into the deep sediments may suggest that the observed increase in dissolved Fe^2+^ at depth is due to organoclastic Fe oxide reduction, i.e. the reduction of Fe oxides coupled to organic matter degradation. However, the presence of Fe oxides is commonly proposed to exert a suppressive effect on methanogenesis because Fe-reducing organisms are able to outcompete methanogens for common substrates (e.g. acetate and hydrogen), reducing the concentration of these primary electron donors to levels that are too low for the growth of CH_4_-producing organisms [70–72]. In addition, Fe oxides may also directly inhibit CH_4_ production due to the capability of methanogens to switch from CH_4_ production to the energetically more favorable reduction of Fe oxides [73–78]. The isotopic composition of pore water CH_4_ in sediments of the Scharendijke basin (Fig 2) indicates a biological origin [79]. Active methanogenesis could therefore indicate limited organoclastic Fe oxide reduction. Recent studies, however, documented that the crystallinity and conductivity of Fe oxides are crucial factors in determining whether CH_4_ production is suppressed or, in fact, stimulated in Fe oxide-rich environments [80–84]. The presence of sedimentary (semi)conductive Fe oxides (e.g. hematite and magnetite) at our study site (Fig 4) could thus potentially allow for concurrent methanogenesis and organoclastic Fe oxide reduction through direct interspecies electron transfer [80–84].

We further propose that the high sediment accumulation rates and, as a consequence, the short residence time of organic matter and Fe oxides in the SO_4_^2-^ reduction zone may provide enough substrate for the concurrent production of CH_4_ and the organoclastic reduction of Fe oxides at depth in the sediment. In highly dynamic organic-rich depositional systems, such as the Scharendijke basin, organoclastic Fe oxide reduction could occur throughout the zone of SO_4_^2-^ reduction and at depth in the methanogenic sediments, with Fe^2+^ only accumulating when the pore waters are depleted in dissolved sulfide.

Interactions between pore water sulfide and deeply buried solid phase Fe oxides could also result in a release of Fe^2+^ into the pore water at depth in the sediment. In this mechanism, Fe oxides enhance the recycling of dissolved sulfide to SO_4_^2-^ in a cryptic S cycle by formation and subsequent disproportionation of elemental S (S_0_) [85]. This SO_4_^2-^ production from re-oxidation of dissolved sulfide with oxidized Fe minerals may thus partly explain the significant SRR (~ 3–20 nmol cm^-3^ d^-1^) measured below the main zone of SO_4_^2-^ reduction at our study site (Fig 2), by stimulating slow rates of organoclastic SO_4_^2-^ reduction and/or SO_4_^2-^-driven AOM [16,25,53,86–90].

A third feasible mechanism for the accumulation of dissolved Fe^2+^ at depth is AOM coupled to Fe oxide reduction (Fe-AOM) [91]:

$${CH}_{4}+8Fe(OH{)}_{3}+15H^{+}\to HCO_{3}^{-}+8{Fe}^{2+}+21H_{2}O$$ (5)

where sedimentary Fe oxides serve as the terminal electron acceptors for the biological oxidation of pore water CH_4_. In theory, the preservation of Fe oxides in the methanogenic sediments as a result of rapid sediment accumulation and thus reduced exposure of solid phase Fe oxides to sulfidic pore waters would allow for such a coupling between CH_4_ oxidation and Fe oxide reduction in the sediments of the Scharendijke basin. Although an increasing body of geochemical evidence indicates that Fe-AOM might be occurring in a variety of different aquatic environments [36,53,92–97], the microbes facilitating these reactions have yet to be identified. The large multi-haem cytochromes (proteins mediating electron transport) in the genomes of one type of methanotrophic archaea known as ANME-2, however, indicate that these organisms should also be able to respire solid Fe oxides through extracellular electron transfer [13–15,98,99]. Based on the available data, identification of a main Fe oxide reduction pathway at depth in the sediment at our study site remains speculative.

### 4.4 Limited CH_4_ removal through AOM

Concentrations of dissolved CH_4_ linearly decrease from around 20 cm depth towards the sediment-water interface, i.e. throughout the zone with high rates of SO_4_^2-^ reduction (Fig 2). Similar overlapping pore water profiles of SO_4_^2-^ and CH_4_ have been observed previously in marine sediments [16–22] and were interpreted as an indication for limited removal of pore water CH_4_ with SO_4_^2-^, which should result in a concave shaped profile of dissolved CH_4_ in the SO_4_^2-^ reduction zone, and potential CH_4_ production above the SMTZ.

The oxidation of CH_4_ coupled to SO_4_^2-^ reduction typically results in a progressive enrichment of the residual upward diffusing CH_4_ in ^13^C-CH_4_ and D-CH_4_ due to the preferential oxidation of isotopically light CH_4_ during AOM [2,79,100,101]. Pore water CH_4_ in sediments of the Scharendijke basin, however, shows no positive excursion towards isotopically enriched CH_4_ in the zone of SO_4_^2-^ reduction (Fig 2). Instead, CH_4_ seems to bypass the SO_4_^2-^ reduction zone without any significant change in δ^13^C-CH_4_ and to escape into the bottom water, where aerobic CH_4_ oxidation results in a shift towards heavier δ^13^C-CH_4_. Note that the small negative excursion in δ^13^C-CH_4_ around 60 cm depth in the sediment could indicate enzyme-mediated equilibrium carbon isotope exchange during AOM at low (< 0.5 mM) SO_4_^2-^ concentrations [102,103]. In this mechanism, SO_4_^2-^ limitation leads to an apparent inverse isotope effect due to an enzyme-level reversibility of AOM, where the relative equilibrium fractionation of the reverse reaction (i.e. AOM back flux) exceeds the forward reaction. Interestingly, the negative ^13^C excursion in pore water CH_4_ coincides with the sulfide penetration depth, suggesting that the production of SO_4_^2-^ from re-oxidation of dissolved sulfide with oxidized Fe minerals could fuel low rates of SO_4_-AOM at this depth.

Instead of exhibiting the characteristic shift towards more δD-enriched CH_4_ commonly observed in the SMTZ, pore water CH_4_ is depleted in δD around the zone of SO_4_^2-^ reduction compared to CH_4_ at depth. Such a negative excursion in δD-CH_4_ may point towards a substrate shift from CO_2_ reduction at depth to acetate fermentation in the SO_4_^2-^ reduction zone, as CH_4_ produced from acetate fermentation is generally more depleted in heavy D isotopes relative to CH_4_ from CO_2_ reduction [79]. This rather unusual observation implies CH_4_ production in the surface sediments characterized by high rates of SO_4_^2-^ reduction. In these sediments, the SO_4_^2-^-reducing bacteria are thought to outcompete methanogens for the available hydrogen necessary for CO_2_ reduction. However, our results suggest that the high burial rates of relatively fresh organic matter may provide enough methanogenic substrates, such as acetate, to allow for concurrent SO_4_^2-^ reduction and acetate fermentation in the surface sediments.

Previous studies have shown that, when co-occurring, CH_4_ production may conceal the isotopic signature of AOM [16,36,104,105]. Methanogenesis in the surface sediments could therefore mask small rates of AOM in the SMTZ. In the model, we allowed for CH_4_ production in the SO_4_^2-^ reduction zone (Table E in S1 File) to estimate the relative contribution of methanogenesis and AOM required to reconstruct the observed pore water profile of CH_4_ around the SMTZ at our study site (Fig 5). Depth-integrated rates of CH_4_ production (~ 0.22 mol CH_4_ m^-2^ yr^-1^) and SO_4_-AOM (~ 0.07 mol CH_4_ m^-2^ yr^-1^) in the upper 20 cm of sediment reveal a ratio of AOM/CH_4_ production of 0.32. Considering the reported range of C isotope fractionation factors (ε_C_) associated with methanotrophy (ε_C_ = 4–30 ‰) and methanogenesis (ε_C_ = 49–95 ‰) [79], such an AOM/CH_4_ production ratio could allow an isotopic balance of CH_4_ production and consumption, i.e. no significant change in δ^13^C-CH_4_, as observed in sediments of the Scharendijke basin.

Based on these results, we conclude that the surface sediments of the Scharendjike basin are most likely characterized by a complex interplay of concurrent SO_4_^2-^ reduction and slow rates of CH_4_ production and consumption, supporting recent findings showing that the traditional concept of a strict dissimilatory respiration sequence is oversimplified and that SO_4_^2-^ reduction, AOM and methanogenesis can co-occur in marine sediments [16,25,53,78,86,87,89,90,104,106].

The modeled rates of SO_4_-AOM (~ 1.4 nmol cm^-3^ d^-1^) are within the range of AOM rates (~ 0.1–3 nmol cm^-3^ d^-1^) reported in sediments of the North Sea [107] and Skagerrak [108], where pore water CH_4_ is efficiently removed within the SMTZ. The small energetic yields and low growth rates of methanotrophic communities [23,26] likely play a key role in the inefficient CH_4_ consumption through AOM observed in the sediments from the Scharendijke basin. In accordance with a recent study in estuarine Baltic Sea sediments [17], we suggest that the CH_4_ oxidizing microorganisms may have difficulties in keeping up with very rapid sediment accumulation in coastal environments. High sediment burial rates reduce the residence time of methanotrophic organisms in the SMTZ. Given the short residence time, the CH_4_ oxidizing microorganisms cannot build-up sufficient biomass to entirely consume the upward diffusing CH_4_ [23].

The relatively slow removal of CH_4_ in the SO_4_^2-^ reduction zone when compared to the CH_4_ production close to the sediment surface at our study site leads to a release of CH_4_ from the sediment into the overlying water column. Diffusive fluxes based on the pore water profiles of CH_4_ (0.2–0.8 mol m^-2^ yr^-1^) are in good agreement with the modeled CH_4_ efflux of ~ 0.5 mol m^-2^ yr^-1^. These CH_4_ efflux rates fall in the range of CH_4_ fluxes reported for brackish coastal regions, but are three orders of magnitude higher than typical CH_4_ fluxes from diffusive marine sediments (Table 1). When compared to marine seep sediments with an advective CH_4_ transport regime in active ocean margin sites, the CH_4_ efflux at our study site falls within the lower range of CH_4_ fluxes reported (Table 1). The CH_4_ efflux from Scharendijke sediments to the water column, however, likely is even higher during the summer months when bottom waters are hypoxic and/or anoxic (e.g. [29,31,109]), thus increasing the potential for CH_4_ to escape to the atmosphere at the end of summer hypoxia [31]. In addition, the high concentrations of CH_4_ at depth in the model (> 30 mM) exceed the CH_4_ solubility concentrations of ~ 10 mM for the environmental conditions at our study site [110]. The presence of free CH_4_ gas and subsequent ebullition could thus further increase the potential CH_4_ loss from the sediments of the Scharendijke basin. More research is needed to quantify CH_4_ emissions during summertime hypoxia and from CH_4_ ebullition in Lake Grevelingen.

**Table 1:** Reported CH_4_ effluxes from brackish and marine sediments in mol m^-2^ yr^-1^.

Area	Salinity	CH_4_ efflux	Reference
Tidal flats	~ 1–26	0.04–8^a^	[32]
Tidal marshes	~ 1–35	0.01–6^a^	[32]
European tidal estuary (Westerschelde Estuary)	~ 1–30	0.04–70^b^	[111]
Mangroves	~ 7–56	0.01–1.9	[32]
Himmerfjärden estuary (Baltic Sea, Sweden)	5–7	0.1–0.8	[17]
Southern Baltic Sea coast (Germany)	7–10	0.02–57^c^	[109]
Gdansk Deep (South eastern Baltic Sea, gassy sediments)	8–12^d^	0.04–1.2^c^	[112]
Cape Lookout Bight (North Atlantic)	~ 34	0.4–23^e^	[29]
Active margins site (including CH_4_ seeps)	~ 34–36	0.04–33	[23]
Passive margins site	~ 34–36	2 * 10^−4^	[23]
Scharendijke basin (Lake Grevelingen, North Sea)	29–32	0.2–0.8^f^	This study

^a^ Excluding freshwater sites (i.e. salinity < 1) and sites affected by sewage;

^b^ fluxes of > 200 mol m^-2^ yr^-1^ are reported for the freshwater endmember;

^c^ values in October are between 0.01 and 0.06 mol m^-2^ yr^-1^, while values for June/July range from 13 to 57 mol m^-2^ yr^-1^, respectively;

^d^ [113];

^e^ Annual range, with fluxes of < 0.5 mol m^-2^ yr^-1^ between November and May, and a peak of 23 mol m^-2^ yr^-1^ in August;

^f^ Data available for October until May. Note that fluxes are likely higher during summer hypoxia (i.e. between June and September).

## 5. Conclusions

The Scharendijke basin, located in a former estuarine channel of Lake Grevelingen, is characterized by high organic matter input to the sediment (~ 91 mol C m^-2^ yr^-1^) and seasonal bottom water hypoxia. Rapid sediment accumulation (~ 13 cm yr^-1^) in this coastal basin results in a high burial efficiency of organic matter past the zone of SO_4_^2-^ reduction (> 78%), thereby fueling CH_4_ production at depth in the sediment. However, unlike in most marine systems studied to date, the upward diffusing CH_4_ is not efficiently removed in the SMTZ, resulting in high CH_4_ effluxes from the sediment into the overlying water column (0.2–0.8 mol m^-2^ yr^-1^). Methane isotope analysis suggests that these high CH_4_ effluxes are due to (acetotrophic) methanogenesis in the surface sediments and the lack of substantial CH_4_ removal in the SMTZ. During summer hypoxia, CH_4_ fluxes from the sediment are likely even higher, which may allow CH_4_ to accumulate below the pycnocline in the water column [31]. Passing summer storms, in particular towards the end of summer hypoxia, could thus result in a release of the CH_4_ to the atmosphere.

Our results indicate that, in rapidly accumulating marine coastal sediments, slow-growing methanotrophic organisms may not be able to build up sufficient biomass to allow for efficient consumption of pore water CH_4_. High organic matter input could further support the co-occurrence of various dissimilatory respiration processes allowing for CH_4_ production in shallow sediments of eutrophic coastal areas. We conclude that anthropogenic eutrophication of coastal systems thus may increase the release of CH_4_ from these sediments.

## Supporting Information

S1 File Additional solid phase data, overview of sampling campaigns and model parameterization. (PDF)
