# Supplementary material for: Rapid Sediment Accumulation Results in High Methane Effluxes from Coastal Sediments
Source: PLoS One. 2016 Aug 25;11(8):e0161609. doi: 10.1371/journal.pone.0161609 (PMC4999275; doi:10.1371/journal.pone.0161609)
Supplement: S1 File — (PDF) [file pone.0161609.s001.pdf]

SUPPORTING INFORMATION

Rapid sediment accumulation results in high methane effluxes from coastal sediments

Matthias Egger*[1], Wytze Lenstra[1], Dirk Jong[1], Filip J. R. Meysman[2,3], Célia J. Sapart[4,5], Carina van der Veen[4], Thomas Röckmann[4], Santiago Gonzalez[6], and Caroline P. Slomp[1]

[1] Department of Earth Sciences – Geochemistry, Faculty of Geosciences, Utrecht University, Utrecht, The Netherlands

[2] Department of Estuarine and Deltaic Studies, Royal Netherlands Institute for Sea Research, Yerseke, The Netherlands

[3] Department of Analytical, Environmental, and Geochemistry, Vrije Universiteit Brussel, Brussels, Belgium

[4] Institute for Marine and Atmospheric Research Utrecht, Utrecht University, Utrecht, The Netherlands

[5] Laboratoire de Glaciologie, Université Libre de Bruxelles, Brussels, Belgium

[6] Department of Marine Microbiology and Biogeochemistry, Royal Netherlands Institute for Sea Research (NIOZ), Texel, The Netherlands

*Corresponding author. E-mail: m.j.egger@uu.nl

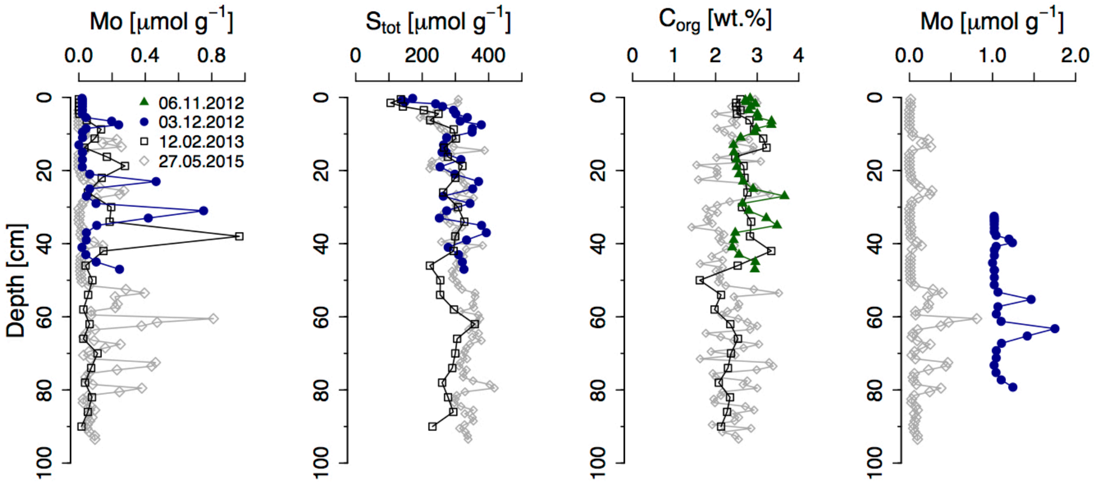

Figure A. Solid phase depth profiles from sediment cores collected between 2012 and 2015 in the Scharendijke basin of Lake Grevelingen. The figure on the right shows the Mo profile for December 2012 (offset by 1 µmol g^{-1} for visualization purposes) relative to the sediment-water interface in May 2015, assuming an average sedimentation velocity of 13 cm yr^{-1} (i.e., a sediment deposition of ~ 32 cm between 3 December 2012 and 27 May 2015).

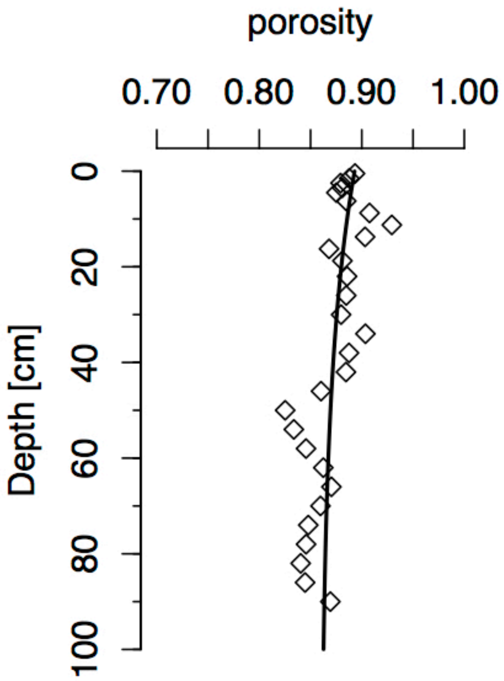

Figure B. Measured (diamonds) and modeled (black line) sediment porosity at Scharendijke basin. Data from February 2013.

Table A. Overview of sampling campaigns in the Scharendijke basin.

	18-10-12	*06-11-12*	*03-12-12*	*12-02-13*	*13-05-13*	*27-05-15*	*10-11-15*
Sampling							
Core tube length[a]	120 cm	60 & 120 cm	60 cm	120 cm	120 cm	120 cm	120 cm
Core slicing	-	**X**	**X**	**X**	-	**X**	-
Rhizon sampling	**X**	**X**	-	-	**X**	-	**X**
Data available							
SO_4^{2-}	**X**	**X**	**X**	**X**	**X**	-	**X**
H_2S	-	**X**	**X**	**X**	-	-	-
CH_4	-	**X**	-	**X**	**X**	-	**X**
Fe^{2+}	**X**	**X**	**X**	**X**	**X**	-	**X**
NH_4^+	**X**	**X**	**X**	**X**	**X**	-	**X**
$\delta^{13}C$-CH_4	-	**X**[b]	-	**X**	-	-	**X**[b]
δD-CH_4	-	**X**[b]	-	**X**	-	-	**X**[b]
SRR[c]	-	-	-	-	-	-	**X**
Tot SP[d]	-	-	**X**	**X**	-	**X**	-
C_{org}	-	**X**	-	**X**	-	**X**	-
Fe speciation	-	-	-	-	-	**X**	-

[a] core diameter was 6 cm for all cores; [b] bottom water sample only; [c] SRR = sulfate reduction rates; [d] total elemental composition of solid phase; Note that samples for CH_4 and SRR were taken from separate pre-drilled cores, while all other pore water and solid phase data were collected from the same sediment core.

Table B. Chemical species included in the model.

Species	Notation	Type
Organic matter[a]	$OM^{\propto,\beta}$	Solid
Iron oxides[a]	$Fe(OH)_3^{\propto,\beta}$	Solid
Iron monosulfide	FeS	Solid
Pyrite	FeS_2	Solid
Elemental sulfur	S_0	Solid
Oxygen	O_2	Solute
Sulfate	SO_4^{2-}	Solute
Iron	Fe^{2+}	Solute
Hydrogen sulfide[b]	$\sum H_2S$	Solute
Methane	CH_4	Solute
Ammonium[b]	$\sum NH_4^+$	Solute
Nitrate	NO_3^-	Solute

[a] There are two types of species: reactive (α) and less reactive (β)

[b] ∑ denotes that all species of an acid are included

Table C. Environmental parameters used in the model

Parameter	Symbol	Value	Units
Porosity at surface	ϕ_0	0.893	-
Porosity at depth	ϕ_∞	0.860	-
Porosity e-folding distance	γ	40	cm
Sediment density	ρ	2.65	g cm^{-3}
Temperature	T	4	^oC
Salinity	S	32	-
Pressure	P	5.5	bar
Tortuosity	θ^2	$1 - 2\ln(\phi)$	-
Molecular diffusion coefficient corrected for tortuosity	D'	$D' = \frac{D_m}{\theta^2}$	cm^{-2} yr^{-1}
Sediment accumulation rate*	F_{sed}	3.767	g cm^{-2} yr^{-1}
Advective velocity at surface	v_0	$\frac{F_{sed}}{\rho(1-\phi_0)}$	cm yr^{-1}
Advective velocity at depth	v_∞	$\frac{F_{sed}}{\rho(1-\phi_\infty)}$	cm yr^{-1}

Table D. Reaction pathways and stoichiometries implemented in the model.

**Primary redox reactions***	
$OM^{\propto,\beta} + aO_2 \rightarrow aCO_2 + bNH_4^+ + aH_2O$	R1
$OM^{\propto,\beta} + \frac{4a}{5}NO_3^- + \frac{4a}{5}H^+ \rightarrow aCO_2 + bNH_4^+ + \frac{2a}{5}N_2 + \frac{7a}{5}H_2O$	R2
$OM^{\propto,\beta} + 4aFe(OH)_3^{\propto} + 12aH^+ \rightarrow aCO_2 + bNH_4^+ + 4aFe^{2+} + 13aH_2O$	R3
$OM^{\propto,\beta} + \frac{a}{2}SO_4^{2-} + aH^+ \rightarrow aCO_2 + bNH_4^+ + \frac{a}{2}H_2S + aH_2O$	R4
$OM^{\propto,\beta} \rightarrow \frac{a}{2}CO_2 + bNH_4^+ + \frac{a}{2}CH_4$	R5
**Secondary redox and other reaction equations†**	
$2O_2 + NH_4^+ + 2HCO_3^- \rightarrow NO_3^- + 2CO_2 + 3H_2O$	R6
$O_2 + 4Fe^{2+} + 8HCO_3^- + 2H_2O \rightarrow 4Fe(OH)_3^{\propto} + 8CO_2$	R7
$2O_2 + FeS \rightarrow SO_4^{2-} + Fe^{2+}$	R8
$7O_2 + 2FeS_2 + 2H_2O \rightarrow 4SO_4^{2-} + 2Fe^{2+} + 4H^+$	R9
$2O_2 + H_2S + 2HCO_3^- \rightarrow SO_4^{2-} + 2CO_2 + 2H_2O$	R10
$2O_2 + CH_4 \rightarrow CO_2 + 2H_2O$	R11
$2Fe(OH)_3^{\propto} + H_2S + 4CO_2 \rightarrow 2Fe^{2+} + S_0 + 4HCO_3^- + 2H_2O$	R12
$2Fe(OH)_3^{\beta} + H_2S + 4CO_2 \rightarrow 2Fe^{2+} + S_0 + 4HCO_3^- + 2H_2O$	R13
$Fe^{2+} + H_2S \rightarrow FeS + 2H^+$	R14
$FeS + H_2S \rightarrow FeS_2 + H_2$	R15
$4S_0 + 4H_2O \rightarrow 3H_2S + SO_4^{2-} + 2H^+$	R16
$FeS + S_0 \rightarrow FeS_2$	R17
$SO_4^{2-} + CH_4 + CO_2 \rightarrow 2HCO_3^- + H_2S$	R18

* Organic matter (OM) is of the form $(CH_2O)_a(NH_4^+)_b$, with 'a'=1 and 'b' = 1/16. Note that both organic matter and Fe oxides are divided into a highly reactive (α) and less reactive (β) phases to account for differences in reactivity and crystallinity between different species. The reaction network is based on [1] and [2].

R6 = nitrification; *R7* = $Fe(OH)_3$ formation; *R8* = FeS oxidation; *R9* = FeS_2 oxidation; *R10* = H_2S oxidation; *R11* = aerobic CH_4 oxidation; *R12* and *R13* = $Fe(OH)_3$ reduction by H_2S; *R14*= FeS formation; *R15* = pyrite formation (H_2S pathway); *R16* = S_0 disproportionation; *R17*= pyrite formation (polysulfide pathway); *R18* = SO_4-AOM.

Table E. Reaction equations implemented in the model.

**Primary redox reaction equations**	
$R_1 = k_{\propto,\beta} OM^{\propto,\beta} \left(\frac{[O_2]}{K_{O_2}+[O_2]}\right)$	(E1)
$R_2 = k_{\propto,\beta} OM^{\propto,\beta} \left(\frac{[NO_3^-]}{K_{NO_3^-}+[NO_3^-]}\right)\left(\frac{K_{O_2}}{K_{O_2}+[O_2]}\right)$	(E2)
$R_3 = k_{\propto,\beta} OM^{\propto,\beta} \left(\frac{[Fe(OH)_3^{\alpha}]}{K_{Fe(OH)_3^{\alpha}}+[Fe(OH)_3^{\alpha}]}\right)\left(\frac{K_{NO_3^-}}{K_{NO_3^-}+[NO_3^-]}\right)\left(\frac{K_{O_2}}{K_{O_2}+[O_2]}\right)$	(E3)
$R_4 = \Psi_{SO4} k_{\propto,\beta} OM^{\propto,\beta} \left(\frac{[SO_4^{2-}]}{K_{SO_4^{2-}}+[SO_4^{2-}]}\right)\left(\frac{K_{Fe(OH)_3^{\alpha}}}{K_{Fe(OH)_3^{\alpha}}+[Fe(OH)_3^{\alpha}]}\right)\left(\frac{K_{NO_3^-}}{K_{NO_3^-}+[NO_3^-]}\right)\left(\frac{K_{O_2}}{K_{O_2}+[O_2]}\right)$	(E4)
$R_5^{\propto} = \Psi_{CH4} k_{\propto} OM^{\propto} \left(\frac{K_{SO_4^{2-}}}{K_{SO_4^{2-}}+[SO_4^{2-}]}\right)\left(\frac{K_{Fe(OH)_3^{\alpha}}}{K_{Fe(OH)_3^{\alpha}}+[Fe(OH)_3^{\alpha}]}\right)\left(\frac{K_{NO_3^-}}{K_{NO_3^-}+[NO_3^-]}\right)\left(\frac{K_{O_2}}{K_{O_2}+[O_2]}\right)$	(E5a)
$R_5^{\beta} = \Psi_{CH4} k_{\beta} OM^{\beta} \left(\frac{K_{Fe(OH)_3^{\alpha}}}{K_{Fe(OH)_3^{\alpha}}+[Fe(OH)_3^{\alpha}]}\right)\left(\frac{K_{NO_3^-}}{K_{NO_3^-}+[NO_3^-]}\right)\left(\frac{K_{O_2}}{K_{O_2}+[O_2]}\right)$	(E5b)*
**Secondary redox and other reaction equations**	
$R_6 = k_1[O_2][NH_4^+]$	(E6)
$R_7 = k_2[O_2][Fe^{2+}]$	(E7)
$R_8 = k_3[O_2][FeS]$	(E8)
$R_9 = k_4[O_2][FeS_2]$	(E9)
$R_{10} = k_5[O_2][\sum H_2S]$	(E10)
$R_{11} = k_6[O_2][CH_4]$	(E11)
$R_{12} = k_7[Fe(OH)_3^{\propto}][\sum H_2S]$	(E12)
$R_{13} = k_8[Fe(OH)_3^{\beta}][\sum H_2S]$	(E13)
$R_{14} = k_9[Fe^{2+}][\sum H_2S]$	(E14)
$R_{15} = k_{10}[FeS][\sum H_2S]$	(E15)
$R_{16} = k_{11}[S_0]$	(E16)
$R_{17} = k_{12}[FeS][S_0]$	(E17)
$R_{18} = k_{13}[SO_4^{2-}][CH_4]$	(E18)

* Note that methanogenesis with the β fraction of organic matter is not inhibited by SO_4^{2-} reduction to allow for CH_4 production in the SO_4^{2-} reduction zone as indicated by the shift towards more D-depleted CH_4 in the surface sediments (Fig 2).

Table F. Reaction parameters used in the model.

Parameter	Symbol	Value	Units	Values given in literature
Decay constant for OM^{α}	k_{α}	1.62	yr^{-1}	0.05-1.62[a,b]
Decay constant for OM^{β}	k_{β}	0.0086	yr^{-1}	0.0086[b]
Limiting concentration of O_2	K_{O2}	0.02	mM	0.001-0.03[c]
Limiting concentration of NO_3^-	K_{NO3-}	0.004	mM	0.004-0.08[c]
Limiting concentration of $Fe(OH)_3$	$K_{Fe(OH)3}$	65	$\mu mol\ g^{-1}$	65-100[c]
Limiting concentration of SO_4^{2-}	K_{SO42-}	1.6	mM	1.6[c]
Attenuation factor for SO_4^{2-} reduction	Ψ_{SO4}	0.101	-	0.00157-0.075[b,d,e]
Attenuation factor for methanogenesis	Ψ_{CH4}	0.0125	-	0.00157-0.075[b,d,e]
Rate constant for reaction *E6*	k_1	10’000	$mM^{-1}\ yr^{-1}$	5’000-39’000[c,d]
Rate constant for reaction *E7*	k_2	140’000	$mM^{-1}\ yr^{-1}$	140’000[c]
Rate constant for reaction *E8*	k_3	300	$mM^{-1}\ yr^{-1}$	300[c]
Rate constant for reaction *E9*	k_4	1	$mM^{-1}\ yr^{-1}$	1[c]
Rate constant for reaction *E10*	k_5	160	$mM^{-1}\ yr^{-1}$	≥ 160[c]
Rate constant for reaction *E11*	k_6	10^4	$mM^{-1}\ yr^{-1}$	10’000’000[c]
Rate constant for reaction *E12*	k_7	10	$mM^{-1}\ yr^{-1}$	8-100[c,e,f]
Rate constant for reaction *E13*	k_8	0.004	$mM^{-1}\ yr^{-1}$	0.95-5.6[e,f]
Rate constant for reaction *E14*	k_9	100	$mM^{-1}\ yr^{-1}$	100-14’800[b,d,e,f]
Rate constant for reaction *E15*	k_{10}	0.0003	$mM^{-1}\ yr^{-1}$	0.0003-3.15[e,f,g]
Rate constant for reaction *E16*	k_{11}	3	yr^{-1}	3[h]
Rate constant for reaction *E17*	k_{12}	0.1	$mM^{-1}\ yr^{-1}$	0.001-7[e,f,h]
Rate constant for reaction *E18*	k_{13}	0.029	$mM^{-1}\ yr^{-1}$	0.14-120[c,e,f]

[a] [3]; [b] [4]; [c] [5]; [d] [6]; [e] [1]; [f] [2]; [g] [7]; [h] [8]

Table G. Model boundary conditions.

Solids (flux at SWI)	Value	Units
F_OM^{α}	59.9	mol m^{-2} yr^{-1}
F_OM^{β}	31.4	mol m^{-2} yr^{-1}
$F\_Fe(OH)_3^{\alpha}$	0.2	mol m^{-2} yr^{-1}
$F\_Fe(OH)_3^{\beta}$	4.1	mol m^{-2} yr^{-1}
F_FeS	0	mol m^{-2} yr^{-1}
F_FeS_2	0	mol m^{-2} yr^{-1}
F_S_0	0	mol m^{-2} yr^{-1}
**Solutes (Bottom water concentrations)**	**Value**	**Units**
$[O_2]$	0.18	mol m^{-3}
$[NO_3^-]$	0	mol m^{-3}
$[SO_4^{2-}]$	25.2	mol m^{-3}
$[Fe^{2+}]$	0	mol m^{-3}
$[H_2S]$	0	mol m^{-3}
$[NH_4^+]$	0	mol m^{-3}
$[CH_4]$	0	mol m^{-3}

For all chemical species, a zero-gradient boundary condition was specified at the bottom of the model domain. SWI = sediment-water interface.

Supporting References

1. Egger M, Kraal P, Jilbert T, Sulu-Gambari F, Sapart CJ, Röckmann T, et al. Anaerobic oxidation of methane alters sediment records of sulfur, iron and phosphorus in Black Sea. Biogeosciences Discuss. 2016; in review. doi:10.5194/bg-2016-64
2. Rooze J, Egger M, Tsandev I, Slomp CP. Iron-dependent anaerobic oxidation of methane in coastal surface sediments: potential controls and impact. Limnol Oceanogr. 2016; doi:10.1002/lno.10275
3. Moodley L, Middelburg JJ, Herman PMJ, Soetaert K, de Lange GJ. Oxygenation and organic-matter preservation in marine sediments: Direct experimental evidence from ancient organic carbon–rich deposits. Geology. 2005;33: 889. doi:10.1130/G21731.1
4. Reed DC, Slomp CP, Gustafsson BG. Sedimentary phosphorus dynamics and the evolution of bottom-water hypoxia: A coupled benthic-pelagic model of a coastal system. Limnol Oceanogr. 2011;56: 1075–1092. doi:10.4319/lo.2011.56.3.1075
5. Wang Y, Van Cappellen P. A multicomponent reactive transport model of early diagenesis : Application to redox cycling in coastal marine sediments. Geochim Cosmochim Acta. 1996;60: 2993–3014. doi:10.1016/0016-7037(96)00140-8
6. Reed DC, Slomp CP, de Lange GJ. A quantitative reconstruction of organic matter and nutrient diagenesis in Mediterranean Sea sediments over the Holocene. Geochim Cosmochim Acta. Elsevier Ltd; 2011;75: 5540–5558. doi:10.1016/j.gca.2011.07.002
7. Rickard D, Luther GW. Kinetics of pyrite formation by the H2S oxidation of iron (II) monosulfide in aqueous solutions between 25 and 125°C: The mechanism. Geochim Cosmochim Acta. 1997;61: 135–147. doi:10.1016/S0016-7037(96)00322-5
8. Berg P, Rysgaard S, Thamdrup B. Dynamic modeling of early diagenesis and nutrient cycling. A case study in an Arctic marine sediment. Am J Sci. 2003;303: 905–955. doi:10.2475/ajs.303.10.905
